# Comparison of Renal Effects of Ezetimibe–Statin Combination versus Statin Monotherapy: A Propensity-Score-Matched Analysis

**DOI:** 10.3390/jcm9030798

**Published:** 2020-03-15

**Authors:** Jaehyun Bae, Namki Hong, Byung-Wan Lee, Eun Seok Kang, Bong-Soo Cha, Yong-ho Lee

**Affiliations:** 1 Department of Internal Medicine, Yonsei University College of Medicine, Seoul 03722, Korea; JANGGU1985@yuhs.ac (J.B.); nkhong84@yuhs.ac (N.H.); BWANLEE@yuhs.ac (B.-W.L.); EDGO@yuhs.ac (E.S.K.); BSCHA@yuhs.ac (B.-S.C.); 2 Institute of Endocrine Research, Yonsei University College of Medicine, Seoul 03722, Korea

**Keywords:** ezetimibe, statin, renal outcome

## Abstract

Neither lowering of blood lipid levels nor treatment with statins definitively improves renal outcomes. Ezetimibe, a non-statin antilipidemic agent, is known to not only decrease blood lipid levels but also reduce inflammatory response and activate autophagy. We evaluated the effect of adding ezetimibe to a statin on renal outcome compared with statin monotherapy by analyzing longitudinal data of 4537 patients treated with simvastatin 20 mg plus ezetimibe 10 mg (S + E) or simvastatin 20 mg alone (S) for more than 180 days. A propensity-score-based process was used to match baseline characteristics, medical history, and estimated glomerular filtration rate (eGFR) between S + E and S groups. Changes in serum creatinine and incidence of renal events, defined as doubling of serum creatinine to ≥1.5 mg/dL or occurrence of end-stage renal disease after the first day of treatment initiation, were compared between the groups. Among 3104 well-matched patients with a median follow-up of 4.2 years, the S + E group showed a significantly lower risk of renal events than the S group (hazard ratio 0.58; 95% CI 0.35-0.95, *P =* 0.032). In addition, the S + E group tended to preserve renal function compared with the S group throughout follow-up, as assessed by serum creatinine changes (*P*-values for time–group interactions <0.001). These data support the beneficial effects on renal function when combining ezetimibe with a statin.

## 1. Introduction

As the world’s population ages and the prevalence of chronic diseases such as hypertension (HTN) and type 2 diabetes mellitus (T2DM) increases, the incidence of chronic kidney disease (CKD) is rising [1,2]. According to a study by the CKDGen Consortium, reduced glomerular filtration rate (GFR) was associated with 52 million disability-adjusted life years (DALYs) and 2.2 million deaths (3.9% of total deaths) in 2013 worldwide [3]. CKD is also recognized as a significant risk factor for cardiovascular disease (CVD) [2], which is the leading cause of death globally. 

Except for the renin–angiotensin system (RAS) blockers, including angiotensin-converting enzyme (ACE) inhibitors and angiotensin receptor blockers (ARB) [4,5,6], and sodium-glucose cotransporter-2 (SGLT-2) inhibitors [7,8], few agents have been proven to protect against the development or progression of CKD. 

Statins are recommended as the drug of choice for lowering low-density lipoprotein (LDL) cholesterol and preventing cardiovascular disease [9]. Because dyslipidemia is considered a risk factor for CKD [10,11], statins have also been presumed to protect kidney function; however, the results of previous studies are inconsistent [12,13,14].

If statin monotherapy fails to reach the LDL cholesterol target, or side effects such as myopathy occur, current guidelines state that other lipid-lowering agents, such as ezetimibe, should be used in combination or used as a replacement [15,16]. As a result, ezetimibe is a frequently prescribed agent. Ezetimibe decreases blood cholesterol levels by inhibiting intestinal absorption of cholesterol [17]. The efficacy of ezetimibe on lowering LDL cholesterol is estimated to be 10%–18% when used alone, with an additional 25% reduction when combined with a statin [15]. Combination therapy also improved other lipid profiles, such as triglycerides (TG), high-density lipoprotein (HDL) cholesterol, and remnant cholesterol particle, compared with statin monotherapy [18,19,20]. We previously reported that ezetimibe–statin combination therapy was associated with better cardiovascular outcomes versus statin monotherapy, via a propensity-score-matched cohort study and meta-analysis [21,22].

In addition to cardiovascular outcomes, ezetimibe could be assumed to have beneficial effects on renal outcomes, because it has been reported to activate autophagy and inhibit inflammatory response [23,24], which are closely linked with CKD [25]. Researchers have investigated whether ezetimibe combined with a statin was more protective for renal function than statin monotherapy, but their results were inconsistent [26,27,28,29]. However, most previous studies were small in size, of short duration, or included unique subjects, such as perioperative patients.

In this study, we conducted a long-term observational cohort study with propensity-score matching, to evaluate the effect of adding ezetimibe to a statin on renal outcomes compared with statin monotherapy.

## 2. Materials and Methods

### 2.1. Study Design and Population

We conducted a retrospective propensity-matched cohort study, using longitudinal data retrieved from electronic records of Severance Hospital, described previously [21]. Patients who satisfied all the following criteria were included: age ≥19 years; available laboratory data, especially for serum creatinine; and treated with simvastatin 20 mg plus ezetimibe 10 mg (S + E) or simvastatin 20 mg alone (S) for more than 180 days between January 2003 and June 2015. The index date was the first day of S + E or S treatment. Subjects with end-stage renal disease (ESRD), malignancy, or liver cirrhosis within 1 year of the index date were excluded.

Baseline data included demographics, body mass index (BMI), comorbidities, smoking status, medications, and laboratory measurements within 6 months of the index date. Comorbidities were identified by International Classification of Diseases 10th revision (ICD-10) diagnosis codes for the following: hypertension (HTN; I10.0, I10.1, I10.9); diabetes mellitus (DM; E10.0–E14.9); unstable angina (I20); myocardial infarction (MI; I21.0-I24.9, I25.2); and non-hemorrhagic stroke (I64, G46.3, G46.4, I63.9, I69.319). Baseline medications were collected from the medical records, using the national insurance drug codes. Laboratory measurements consisted of serum creatinine, total cholesterol (TC), TG, HDL cholesterol, LDL cholesterol, C-reactive protein (CRP), uric acid, fasting serum glucose, qualitative test for urinary protein, and urinary albumin-to-creatinine ratio.

Serum creatinine levels and the occurrence of newly diagnosed-ESRD (identified by ICD-10 codes E10.22, E11.2, E11.22, E12.22, E13.22, E14.22, I12.0, N18.0, and N18.5) were recorded at the time of each follow-up visit or hospital admission. Serum creatinine levels were determined by using a Hitachi 7600-110 automated chemistry analyzer (Hitachi, Ltd., Tokyo, Japan) and an enzymatic method (CREA, Roche Diagnostics, Indianapolis, IN, USA). The estimated glomerular filtration rate (eGFR) was calculated via the Modification of Diet in Renal Disease (MDRD) study equation. 

The study protocol was approved by the institutional review board of Severance Hospital (No. 4-2018-0852), who waived written informed consent based on retrospective access to existing coded data for research purposes. 

### 2.2. Study Outcomes

The primary outcome measure of this study was “renal event”, defined as the doubling of serum creatinine to ≥1.5 mg/dL or the occurrence of ESRD after the index date. “Doubling of serum creatinine” was a greater than two-fold increase in serum creatinine from baseline. Subjects were followed until the occurrence of an incident renal event, loss to follow-up, death, or 20 March 2016. Renal events that occurred within 3 months after the index date were excluded from the analysis, because 3 months was considered inadequate to reflect the influence of the study medication(s).

### 2.3. Statistical Analysis

Treatment groups were compared for long-term renal events according to lipid lowering agents. To compare continuous and categorical variables of the S + E and S groups at baseline, Student’s *t* and χ^2^ tests were performed. Propensity-score matching was used to minimize the influence of potential confounding factors (Supplementary Figure S1).

Average serum creatinine levels in both groups during follow-up were described graphically, and autoregressive linear mixed models were used to assess time × group interactions. Survival curves for the cumulative incidence of renal events were estimated by the Kaplan–Meier method and compared via log-rank test. 

To evaluate between-group differences in renal events, Cox proportional-hazards regression models were used, and adjusted hazard ratios (HRs) and 95% confidence intervals (CIs) were calculated and presented. All models were adjusted for age, sex, BMI, history of HTN and DM, smoking status, ACE inhibitor or ARB medication, LDL cholesterol, and eGFR at baseline. We also conducted subgroup analyses according to age, sex, BMI, CKD stage determined by eGFR category, history of HTN, and DM. The *P*-value for interaction between a subgroup variable and renal outcome was assessed in each subgroup analysis.

Continuous variables are expressed as means ± standard deviation (SD), and categorical variables are expressed as numbers (percentages, %). A two-sided *P*-value <0.05 was considered statistically significant. Statistical analyses were performed by using SPSS version 21.0 for Windows (IBM Corp., Armonk, NY, USA).

## 3. Results

### 3.1. Study-Population Characteristics

A total of 4537 patients who were treated with S + E (*n* = 2895) or S (*n* = 1642) for more than 180 days were included in the present study (Supplementary Figure S2). The two groups displayed different characteristics at baseline (Supplementary Table S1). The S + E group was composed of younger patients with higher BMI, fasting serum glucose, and eGFR levels; lower total cholesterol, LDL cholesterol, and HDL cholesterol levels; a more prevalent history of current smoking, diabetes, and stroke; and less history of HTN compared with the S group. More patients in the S group were taking β-blocker, calcium channel blocker (CCB), ACE inhibitors, or ARB compared to the S + E group.

After 1:1 propensity-score matching, there were 3104 well-matched patients (Table 1). The average age of S + E and S groups was 68.3 and 68.8 years, respectively. Approximately half of the subjects were men (766 (49.4%) in S + E and 797 (51.4%) in S), and the baseline eGFR levels were extremely well-matched (76.6 vs. 76.7 mL/min per 1.73 m^2^, respectively; *P* = 0.953). CKD stage was evenly distributed between the two groups. The proportion of patients with HTN in each group was about 49% (758 (48.8%) vs. 755 (48.6%), *P* = 0.914), whereas approximately one-quarter of patients had DM (388 (25.0%) vs. 357 (23.0%), *P* = 0.193). After matching, there were no significant between-group differences for baseline variables, including BMI, β-blocker, CCB, ACE inhibitors, ARB, laboratory parameters associated with glucose or lipid metabolism, history of stroke, or smoking. 

### 3.2. Effects of S + E or S on Changes of Serum Creatinine, LDL-Cholesterol, and CRP

**Figure 1:**
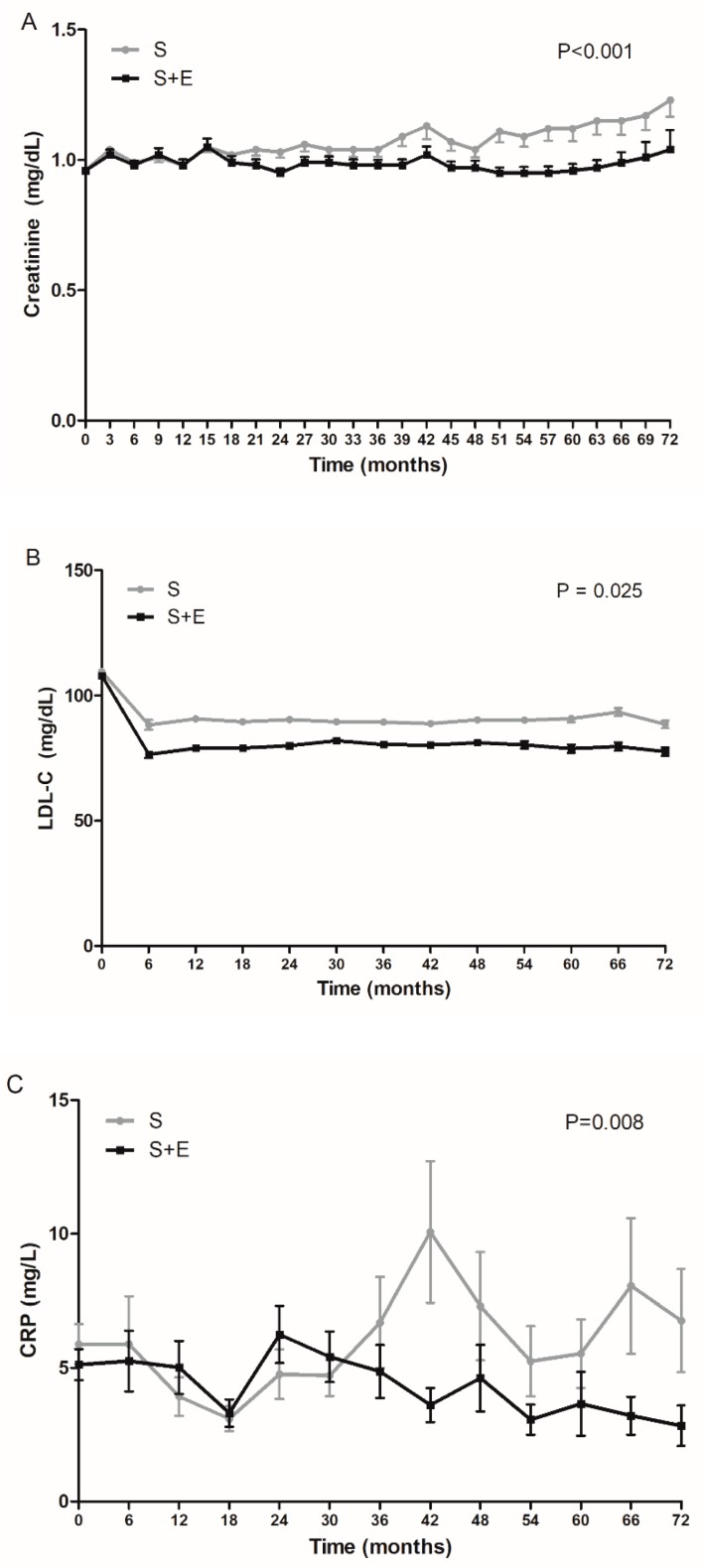
Mean levels of serum creatinine, LDL-cholesterol, and CRP during follow-up by treatment group. (**A**) Serum creatinine, (**B**) LDL-cholesterol, and (**C**) CRP. *P*-value is for time × group interaction by linear mixed model. Error bars represent the standard error of the mean (SEM). S, simvastatin; S + E, simvastatin + ezetimibe.

The median duration of follow-up was 4.2 (IQR: 2.5–6.1) years. Average serum creatinine values gradually increased during follow-up, although there were some fluctuations (Figure 1A). The S + E group showed less of an increase in serum creatinine than the S group. When we evaluated the influence of medication on the creatinine–time curve through linear mixed-model analysis, the results confirmed that adding ezetimibe to simvastatin was the significant factor (*P*-values for time–group interactions <0.001). 

LDL-cholesterol levels were further improved in the S + E group, as expected (Figure 1B). In addition, although there were some fluctuations, patients in the S + E group showed lower CRP levels over time (Figure 1C).

### 3.3. Effects of S + E or S on Occurrence of Renal Events

Among 4537 patients in the initial cohort, renal events occurred in 108 (2.4%), with doubling of serum creatinine to at least 1.5 mg/dL in 97 (2.1%) patients and new-onset end-stage renal disease (ESRD) in 15 (0.3%) patients, with 4 (0.1%) patients experiencing both events. In 3104 propensity-score-matched patients, renal events occurred in 84 (2.7%) patients, 22 of 1552 (1.4%) in the S + E group, and 62 of 1552 (4.0%) patients in the S group. Doubling of serum creatinine occurred in 20 (1.3%) of S + E patients and in 57 (3.7%) S patients, while newly developed ESRD occurred in two (0.1%) of S + E patients and eight (0.5%) S patients. In the S group, three (0.2%) patients were diagnosed with ESRD while the serum creatinine level was doubled.

**Figure 2:**
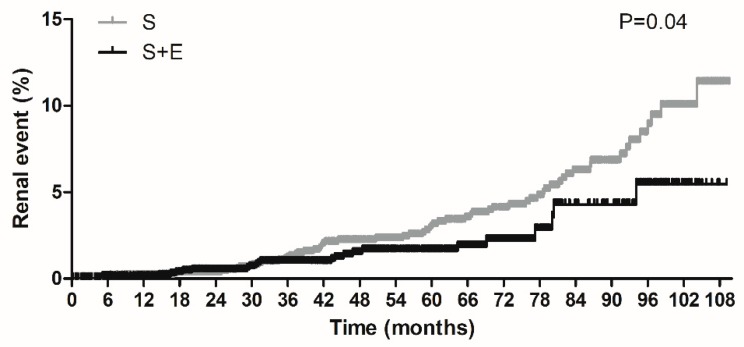
Kaplan–Meier analysis of cumulative incidence of renal events. *P*-value is from log-rank test. S, simvastatin; S + E, simvastatin + ezetimibe.

Using Kaplan–Meier, we observed the renal events were significantly decreased in the S + E group compared with the S group (*P* = 0.038 by log-rank test; see Figure 2). Overall, the S + E group showed a significantly lower risk of renal events than the S group in an unadjusted Cox proportional-hazards regression model (S + E group HR: 0.60, 95% CI: 0.36–0.98, *P* = 0.039) and a multivariate regression model (HR: 0.58, 95% CI: 0.35–0.95, *P* = 0.032) (Table 2). Subgroup analyses demonstrated that risk of renal events tended to be lower in S + E treated patients with older age (≥ 60 years; HR: 0.52, 95% CI: 0.30–0.91, *P* = 0.021), lower BMI (< 25 kg/m^2^; HR: 0.46, 95% CI: 0.24–0.89, *P* = 0.021), preserved renal function (CKD stages 1 and 2; HR: 0.27, 95% CI: 0.10–0.70, *P* = 0.007), no history of HTN (HR: 0.40, 95% CI: 0.16–0.99, *P* = 0.048), or DM (HR: 0.44, 95% CI: 0.21–0.92, *P* = 0.029). However, interaction tests were not statistically significant (*P* for interaction: 0.310 between treatment group and age, 0.326 between treatment group and BMI, 0.073 between treatment group and CKD (categorized by stage 1/2 or stage 3/4), 0.374 between treatment group and history of HTN, and 0.175 between treatment group and history of DM).

## 4. Discussion

In this propensity-score-matched analysis, we compared long-term change in serum creatinine and occurrence of adverse renal events between patients treated with simvastatin + ezetimibe versus simvastatin alone. When we analyzed long-term incidence of renal events, defined as doubling of serum creatinine or occurrence of ESRD, the addition of ezetimibe to a statin showed a significantly lower risk compared with statin monotherapy (HR 0.58, CI: 0.35–0.95, *P* = 0.032). In terms of longitudinal change in serum creatinine, S + E tended to preserve renal function better than S (*P*-values for time–group interactions <0.001).

Risk factors for developing CKD are similar to those for cardiovascular disease (CVD), such as age, HTN, and DM [30]. Renal function decreases with age; an epidemiological study in Turkey showed that prevalence of CKD increases with age in 10-year increments [31]. HTN is also a conventional risk factor for both CKD and ESRD [32]. According to the Multiple Risk Factor Intervention Trial (MRFIT) in the US, the adjusted relative risk for ESRD increased as the stage of HTN worsened [33]. DM is the most common cause of CKD and ESRD in the majority of countries globally [34,35]. For a newly diagnosed T2DM patient without proteinuria, the 20-year risk of nephropathy is estimated to be 41% [36]. Similar to the previously mentioned well-established risk factors, dyslipidemia is considered an aggravating factor for CKD. In several animal studies, which mainly used a high-cholesterol diet, dyslipidemia emerged as a risk factor for kidney injury [37,38,39]. In addition, previous epidemiologic studies have reported that dyslipidemia has a significant association with the risk of kidney disease [10,11,40].

However, there is still controversy regarding whether lowering blood lipid level definitively protects renal function. A meta-analysis published in 2001 reported a favorable effect of lipid-lowering therapy on worsening of eGFR in patients with renal insufficiency [41]. Another meta-analysis in 2006 also suggested that lowering LDL cholesterol via statin therapy had a protective effect for kidney function, presented as reducing proteinuria or loss of eGFR [12]. However, in 2014, a large-scale randomized placebo-controlled study including a wide range of patients with CKD showed that, regardless of CVD history, lowering LDL cholesterol by treatment with a statin plus ezetimibe did not slow the progression of CKD [42].

In addition to lowering LDL cholesterol, statins have other known beneficial effects, such as increasing endothelial nitric oxide (NO) production [43,44] and decreasing vascular resistance [45]. These effects might be protective for the kidneys, but the results of previous studies were controversial. A post hoc analysis of three randomized double-blind controlled trials in 2005 reported renal benefit of statins in patients with previous CVD or at high risk of CVD [46]. Subgroup analysis of the Greek Atorvastatin and Coronary Heart Disease Evaluation (GREACE) study and the Treating to New Targets (TNT) study also showed that statins improved renal function in patients with coronary heart disease [47,48]. However, in a large-scale clinical trial, statin treatment failed to protect kidney function better than non-statin treatment in CKD patients with dyslipidemia [14]. Recently, an umbrella review of meta-analyses was performed, which concluded that the effect of statins on CKD progression is still uncertain [13].

Ezetimibe is the most commonly recommended and used non-statin agent as second-line or as combination therapy with a statin [49]. Previous studies have investigated whether adding ezetimibe to statin treatment is more beneficial to the kidneys than statin alone. Similar to studies assessing the relationship between lowering LDL cholesterol and renal function, the results of these studies were also controversial. Small-scale open-label randomized studies conducted by Japanese researchers reported that adding ezetimibe to Fluvastatin or Pitavastatin produced significant improvement in proteinuria or eGFR changes for 12 or 6 months [26,27]. However, another open-labeled randomized 12-month trial of 286 patients with dyslipidemia showed no significant difference in serum creatinine and albuminuria changes between the statin uptitration group and the statin + ezetimibe group [29]. In a study of patients undergoing vascular surgery, rosuvastatin did not prevent gradual deterioration in renal function, and adding ezetimibe to rosuvastatin was also not renoprotective [28]. Previous studies of ezetimibe had substantial limitations, as they included small populations or patients with specific diseases, with a short (≤1 year) follow-up period. Thus, studies comparing the efficacy of ezetimibe plus a statin with statin monotherapy in terms of renal function are still insufficient. The present study investigated whether adding ezetimibe to a statin is more renoprotective than statin monotherapy among a large group of patients (1552 × 2 well-matched pairs) and with a long (median 4.2 years) follow-up period. To our knowledge, this is the first large-scale long-term study to undertake this comparison. 

It has been reported that ezetimibe confers additional beneficial effects other than lowering blood lipid levels. In animal models, ezetimibe reduced inflammatory response [50,51,52] and promoted autophagy, which resulted in neuroprotection or improvement of nonalcoholic fatty liver disease (NAFLD) [24,53] via AMP protein kinase (AMPK) activation and inhibition of NLR family pyrin domain containing 3 (NLRP3) inflammasome [23]. In addition, ezetimibe is known to activate nuclear factor erythroid 2-related factor 2 (Nrf2) and to reduce oxidative stress in animal models [54]. A small-scale clinical study performed in Japan reported that ezetimibe treatment for six months decreased serum or urinary markers related to atherosclerosis and oxidative stress and ameliorated renal injury in nondiabetic patients with CKD [55].

These findings suggest that ezetimibe could also have beneficial effects on the kidneys, because inflammation, impaired autophagy, and oxidative stress are closely associated with acute or chronic kidney injury [56,57]. Considering that proximal tubules require large amounts of energy for reabsorption processes and have abundant mitochondria, endoplasmic reticulum, and ribosomes, the reduction of oxidative stress and promotion of autophagy (or mitophagy) might play an important role in maintenance of renal function. In addition, plentiful studies have revealed an association between kidney diseases and inflammation, especially the abovementioned NLRP3 inflammasome [58,59,60,61]. We hypothesized that the renoprotective effect of ezetimibe in this study might be due to these mechanisms.

Our study has several limitations. Due to the retrospective design, individuals were not randomly assigned to different treatment groups. It might also have been influenced by potential confounding factors. However, by using propensity-score matching, we aimed to minimize the interference of other characteristics. The lack of follow-up data relative to changes in proteinuria was another limitation, although the reliability of proteinuria as a surrogate marker for renal outcome is also debatable [62,63]. We also could not identify and compare the safety aspects, such as levels of side effects between two groups, because of the limitation of retrospective observational study. We assumed that the side effects and tolerability were similar between two groups, as reported in the “Improved Reduction of Outcomes: Vytorin Efficacy International Trial” (IMPROVE-IT) [64]. In addition, we could not follow up whether patients changed therapy throughout the whole study period. This type of limitation often occurs in large-scale retrospective cohort studies, and is not expected to favorably or adversely influence a particular group. Finally, this study may not be applicable to statins other than simvastatin, especially for the high-intensity statins and their combination therapy with ezetimibe. Future studies including other types of statins are needed. 

In conclusion, in this large-scale longitudinal cohort study, our results showed that adding ezetimibe to a statin resulted in a significantly lower incidence of adverse renal events than statin monotherapy. In addition, a statin combined with ezetimibe tended to preserve renal function relative to serum creatinine change over time. Prospective randomized controlled trials are needed to support our findings and to conduct further research addressing the mechanism of action and effects of ezetimibe on renal function.

## Figures and Tables

**Table 1:** Baseline characteristics by treatment group, after propensity-score matching.

	S + E (*N* = 1552)	S (*N* = 1552)	*P*-Value
Age, years	68.3 ± 10.1	68.8 ± 11.0	0.185
Men, *n* (%)	766 (49.4)	797 (51.4)	0.266
BMI, kg/m^2^	24.5 ± 3.0	24.5 ± 3.2	0.654
Current smoker, *n* (%)	240 (15.5)	217 (14.0)	0.244
Obesity, *n* (%) ^1^	605 (39.0)	617 (39.8)	0.659
HTN, *n* (%)	758 (48.8)	755 (48.6)	0.914
DM, *n* (%)	388 (25.0)	357 (23.0)	0.193
CKD stage 1, *n* (%) ^2^	497 (32.0)	526 (33.9)	0.268
CKD stage 2, *n* (%) ^3^	469 (30.2)	439 (28.3)	0.237
CKD stage 3, *n* (%) ^4^	547 (35.2)	548 (35.3)	0.970
CKD stage 4, *n* (%) ^5^	39 (2.5)	39 (2.5)	>0.999
History of UA, *n* (%)	94 (6.1)	81 (5.2)	0.312
History of MI, *n* (%)	53 (3.4)	54 (3.5)	0.922
History of Stroke, *n* (%)	133 (8.6)	119 (7.7)	0.358
Medication of aspirin, *n* (%)	994 (64.0)	970 (62.5)	0.372
Medication of β-blocker, *n* (%)	641 (41.3)	640 (41.2)	0.971
Medication of CCB, *n* (%)	670 (43.2)	683 (44.0)	0.638
Medication of ACEi/ARB, *n* (%)	724 (46.6)	737 (47.5)	0.640
Proteinuria, *n* (%) ^6,7^	44 (7.1)	59 (8.4)	0.355
Albumin to creatinine ratio (mg/g)	159.9 ± 493.1	238.7 ± 754.2	0.315
Total cholesterol, mg/dL	187.4 ± 50.1	188.9 ± 50.8	0.406
LDL-C, mg/dL	107.9 ± 43.2	109.5 ± 41.6	0.288
HDL-C, mg/dL	49.0 ± 12.8	49.4 ± 12.2	0.341
Triglyceride, mg/dL	145.5 ± 101.0	143.8 ± 86.4	0.620
Uric acid, mg/dL ^8^	5.2 ± 1.4	5.1 ± 1.4	0.103
CRP, mg/L ^9^	5.1 ± 16.9	5.9 ± 17.6	0.418
Fasting glucose, mg/dL	108.9 ± 30.7	107.8 ± 30.6	0.300
HbA1c, % ^10^	7.1 ± 1.6	7.0 ± 1.4	0.437
Creatinine, mg/dL	1.0 ± 0.3	1.0 ± 0.3	0.661
eGFR, mL/min per 1.73 m^2 11^	76.6 ± 31.5	76.7 ± 30.9	0.953

Continuous variables expressed as means ± standard deviation (SD); categorical variables expressed as number (percent). *P* < 0.05 denotes statistical significance. Abbreviations: S, simvastatin; S + E, simvastatin + ezetimibe; BMI, body mass index; HTN, hypertension; DM, diabetes mellitus; CKD, chronic kidney disease; UA, unstable angina; MI, myocardial infarction; CCB, calcium channel blocker; ACE, angiotensin converting enzyme; ARB, angiotensin receptor blocker; LDL-C, low-density lipoprotein cholesterol; HDL-C, high-density lipoprotein cholesterol; CRP, C-reactive protein; HbA1c, glycated hemoglobin; eGFR, estimated glomerular filtration rate. ^1^ Defined as BMI ≥ 25 kg/m^2^. ^2^ Defined as eGFR ≥ 90 mL/min per 1.73 m^2^. ^3^ Defined as 60 ≤ eGFR < 90 mL/min per 1.73 m^2^. ^4^ Defined as 30 ≤ eGFR < 60 mL/min per 1.73 m^2^. ^5^ Defined as 15 ≤ eGFR < 30 mL/min per 1.73 m^2^. ^6^ Defined as qualitative urine protein test ≥ 1+. ^7^ Relatively small number of evaluable patients (S + E, 623; S, 700). Calculated as patients with proteinuria/patients with urinalysis. ^8^ Relatively small number of evaluable patients (S + E, 140; S, 118). ^9^ Relatively small number of evaluable patients (S + E, 843; S, 550). ^10^ Relatively small number of evaluable patients (S + E, 144; S, 228). ^11^ Calculated using MDRD equation.

**Table 2:** Adjusted hazard ratios (HR) for renal events in propensity-score-matched groups.

	Renal Events				
	Events/Total, *n*		HR	95% CI	*P*-Value
	S + E	S			
Overall	**22/1552**	**62/1552**	**0.58**	**0.35–0.95**	**0.032**
Age, years					* 0.310
<60	5/293	8/297	1.14	0.23–5.79	0.868
**≥60**	**17/1259**	**54/1255**	**0.52**	**0.30–0.91**	**0.021**
Sex					* 0.603
Male	13/766	36/797	0.60	0.31–1.15	0.125
Female	9/786	26/755	0.53	0.24–1.13	0.100
BMI					* 0.326
**<25**	**12/947**	**40/935**	**0.46**	**0.24–0.89**	**0.021**
≥25	10/605	22/617	0.69	0.32–1.51	0.356
CKD					* 0.073
**Stage 1/2**	**5/966**	**29/965**	**0.27**	**0.10–0.70**	**0.007**
Stage 3/4	17/586	33/587	0.89	0.48–1.66	0.713
HTN					* 0.374
**No**	**6/794**	**25/797**	**0.40**	**0.16–0.99**	**0.048**
Yes	16/758	37/755	0.71	0.39–1.29	0.254
DM					* 0.175
**No**	**9/1164**	**36/1195**	**0.44**	**0.21–0.92**	**0.029**
Yes	13/388	26/357	0.73	0.37–1.46	0.372

Abbreviations: S, simvastatin; S + E, simvastatin + ezetimibe; BMI, body mass index; CKD, chronic kidney disease; HTN, hypertension; DM, diabetes mellitus; HR, hazards ratio; CI, confidence interval. Cox regression model was adjusted for age, sex, BMI, DM, HTN, low-density lipoprotein cholesterol at baseline, estimated glomerular filtration rate at baseline, current smoking, use of ACE inhibitors/angiotensin receptor blockers at baseline. * *P*-values for interaction between treatment group and variables. *P* < 0.05 (bolded) denotes statistical significance.

## Supplementary Materials

The following are available online at https://www.mdpi.com/2077-0383/9/3/798/s1. Figure S1: Standardized mean difference and absolute standardized difference for covariates before and after propensity matching of patients treated with simvastatin + ezetimibe or simvastatin alone. (A) Standardized mean difference; (B) absolute standardized difference. Figure S2: The flowchart of the retrospective cohort study. Table S1: Baseline characteristics of treatment groups before propensity-score matching.
