# Supplementary material for: Comparison of Renal Effects of Ezetimibe–Statin Combination versus Statin Monotherapy: A Propensity-Score-Matched Analysis
Source: J Clin Med. 2020 Mar 15;9(3):798. doi: 10.3390/jcm9030798 (PMC7141215; doi:10.3390/jcm9030798)
Supplement: Supplementary file 1 [file jcm-09-00798-s001.pdf]

Figure S1. Standardized mean difference and absolute standardized difference for covariates before and after propensity matching of patients treated with simvastatin+ezetimibe or simvastatin alone. (A) standardized mean difference, (B) absolute standardized difference

A

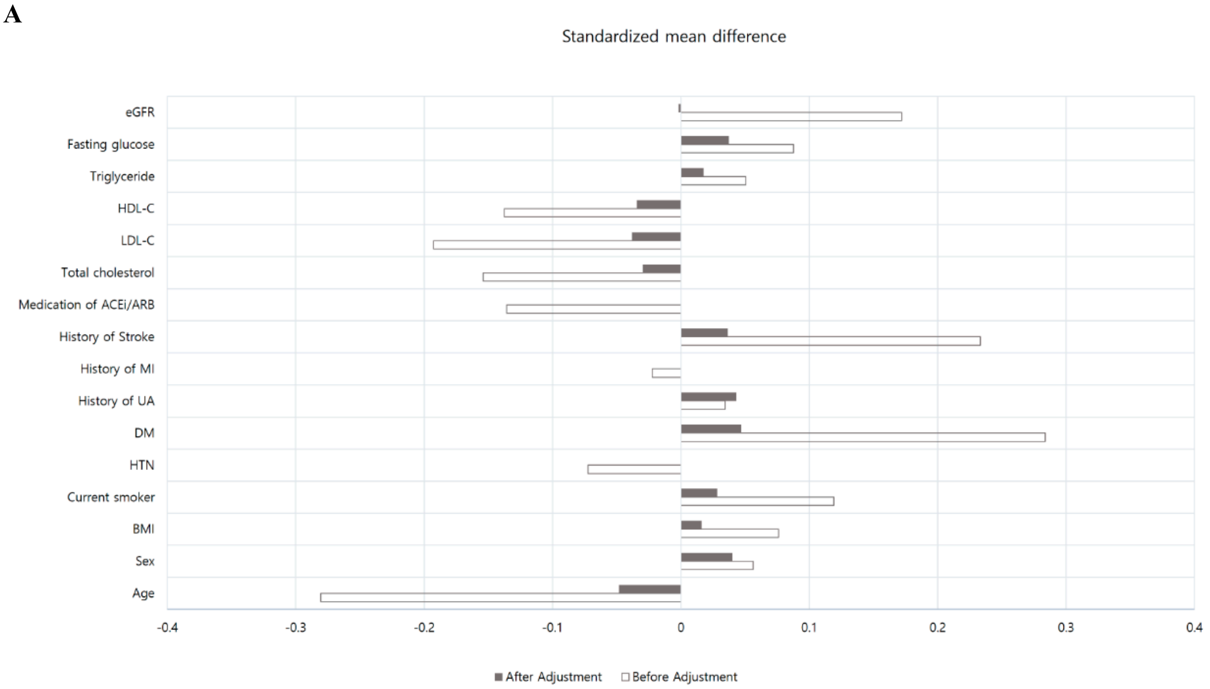

B

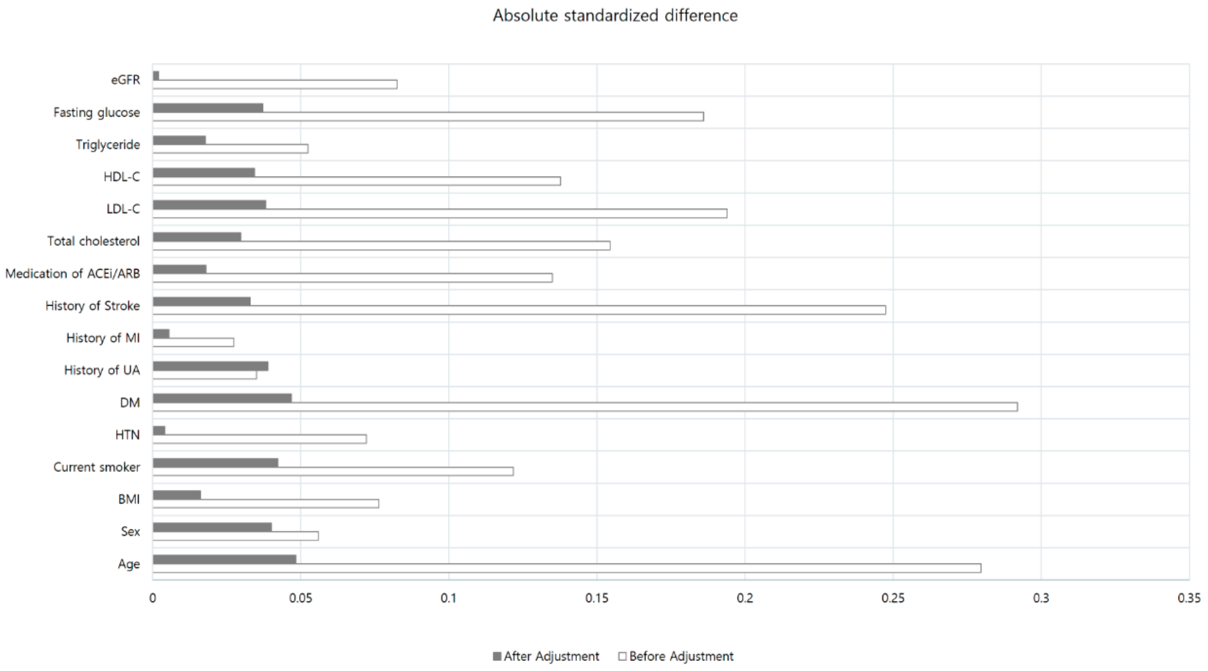

eGFR, estimated glomerular filtration rate; HDL-C, high density lipoprotein cholesterol; LDL-C, low density lipoprotein cholesterol; ACEi/ARB, angiotensin converting enzyme inhibitor or angiotensin receptor blocker; MI, myocardial infarction; UA, unstable angina; DM, diabetes mellitus; HTN, Hypertension; BMI, body mass index.

Figure S2. The flow chart of the retrospective cohort study

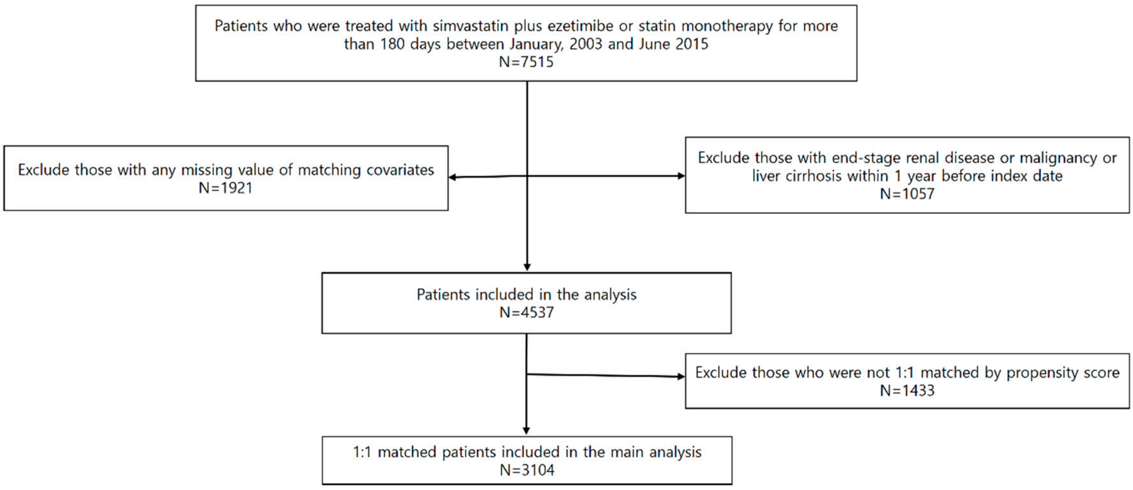

Table S1. Baseline Characteristics of Treatment Groups Before Propensity Score Matching

	S+E (N=2895)	S (N=1642)	P-value
Age, years	66.4 ± 10.5	69.4 ± 11.2	<0.001
Men, n (%)	1419 (49.0)	851 (51.8)	0.07
BMI, kg/m^2	24.7 ± 3.1	24.5 ± 3.2	0.01
Current smoker, n (%)	513 (17.7)	219 (13.3)	<0.001
Obesity n, (%)[1]	1212 (41.9)	650 (39.6)	0.13
HTN n, (%)	1329 (45.9)	813 (49.5)	0.02
DM n, (%)	1006 (34.7)	357 (21.7)	<0.001
CKD stage 1, n (%)[2]	1085 (37.5)	533 (32.5)	0.001
CKD stage 2, n (%)[3]	840 (29.0)	465 (28.3)	0.62
CKD stage 3, n (%)[4]	901 (31.1)	605 (36.8)	<0.001
CKD stage 4, n (%)[5]	69 (2.4)	39 (2.4)	0.99
History of UA, n (%)	171 (5.9)	84 (5.1)	0.27
History of MI, n (%)	94 (3.2)	60 (3.7)	0.47
History of Stroke, n (%)	430 (14.9)	119 (7.2)	<0.001
Medication of aspirin, n (%)	1794 (62.0)	1030 (62.7)	0.61
Medication of β-blocker, n (%)	1051 (36.3)	691 (42.1)	<0.001
Medication of CCB, n (%)	1176 (40.6)	720 (43.8)	0.03
Medication of ACEi/ARB, n (%)	1210 (41.8)	797 (48.5)	<0.001
Proteinuria,n (%)[6,7]	88 (8.5)	66 (8.8)	0.79
Total cholesterol, mg/dL	181.9 ± 50.9	189.8 ± 50.7	<0.001
LDL-C, mg/dL	102.4 ± 42.4	110.5 ± 41.6	<0.001
HDL-C, mg/dL	47.9 ± 12.2	49.6 ± 12.3	<0.001
Triglyceride, mg/dL	149.1 ± 111.2	143.9 ± 86.1	0.08
Fasting glucose, mg/dL	110.1 ± 30.6	107.4 ± 30.0	0.005
HbA1c, %[8]	7.1 ± 1.5	6.9 ± 1.4	0.24
Creatinine, mg/dL	0.9 ± 0.3	1.0 ± 0.3	<0.001
eGFR, mL/min per 1.73m^2 [9]	81.4 ± 33.8	75.7 ± 30.6	<0.001

Continuous variables expressed as means + standard deviation (SD); categorical variables expressed as number (percent).

Abbreviations: S, simvastatin; S+E, simvastatin+ezetimibe; BMI, body mass index; HTN, hypertension; DM, diabetes mellitus; CKD, chronic kidney disease; UA, unstable angina; MI, myocardial infarction; CCB, calcium channel blocker; ACE, angiotensin converting enzyme; ARB, angiotensin receptor blocker; LDL-C, low density lipoprotein cholesterol; HDL-C, high density lipoprotein cholesterol; HbA1c, glycated hemoglobin; eGFR, estimated glomerular filtration rate.

[1] Defined as BMI $\geq$ 25 kg/m^2

[2] Defined as eGFR $\geq$ 90 mL/min per 1.73m^2

[3] Defined as 60 $\leq$ eGFR < 90 mL/min per 1.73m^2

[4] Defined as 30 $\leq$ eGFR < 60 mL/min per 1.73m^2

[5] Defined as 15 $\leq$ eGFR < 30 mL/min per 1.73m^2

[6] Defined as qualitative urine protein test $\geq$ 1+

[7] Relatively small number of evaluable patients (S+E, 1038; S, 747). Calculated as patients with proteinuria/patients with urinalysis

[8] Relatively small number of evaluable patients (S+E, 323; S, 238).

[9] Calculated using MDRD equation.
